# Primary (congenital) occipital encephalomeningocele in a newborn born to a mother despite folic acid supplementation

**DOI:** 10.1002/ccr3.7601

**Published:** 2023-07-04

**Authors:** Binod Paudel, Gopal K. Yadav

**Affiliations:** ^1^ Department of Internal Medicine Grahun Primary Hospital, Ministry of Health and Population Waling Nepal; ^2^ Department of Internal Medicine Kalaiya Hospital, Ministry of Health and Population Kalaiya Nepal

**Keywords:** folic acid, meningoencephalocele, neural tube defects, pregnancy

## Abstract

**Key Clinical Message:**

The etiology of neural tube defects is multifactorial, with a wide interplay of genetic and environmental factors. However, periconceptional folic acid should be supplemented in antenatal care.

**Abstract:**

We described a case of neural tube defects (NTDs), specifically occipital encephalomeningocele, in a child born to a mother who received folic acid supplementation. A wide interplay of genetic and environmental factors exists in its causation. Although folic acid confers advantage, the relationship with the causation of NTDs still remains unclear.

## Graphical abstract

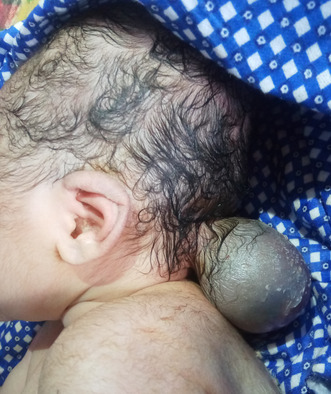

## 1 DESCRIPTION OF IMAGE

**FIGURE 1:**
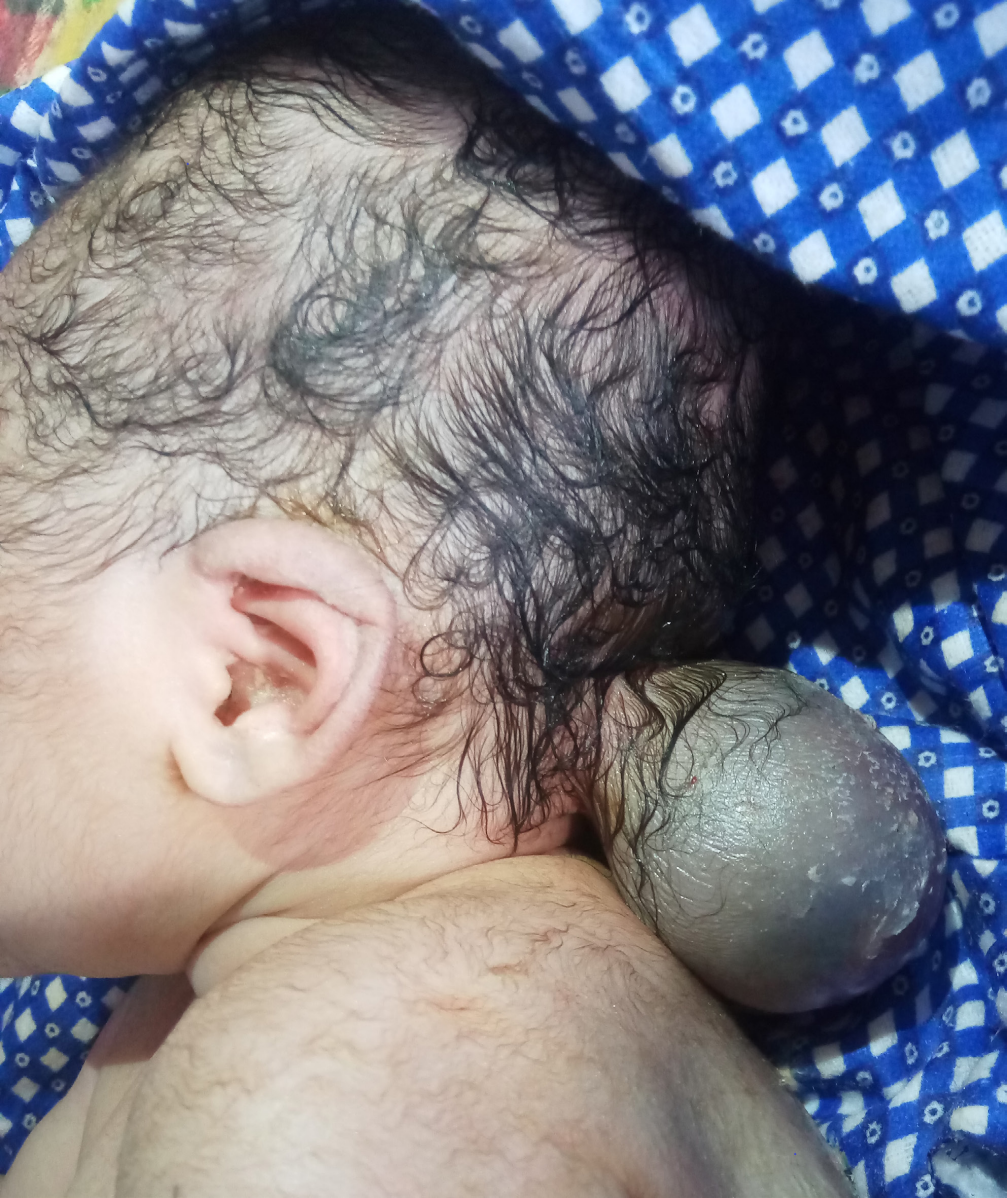
Occipital encephalomeningocele.

A newborn female baby weighing 2900 g was born via spontaneous vaginal delivery to a 17‐year primigravida female. At birth, the baby was noted to have a bulging from the posterior part of the head (Figure 1). The mother had been supplementing with folic acid (0.4 mg/day) for 1 month prior to pregnancy and throughout the first trimester. Prenatal screening at 18 weeks of gestation was unremarkable on obstetric scan.

These neural tube defects (NTDs) occurred due to failure of neural tube closure. The etiology of NTDs is multifactorial, with a wide interplay of genetic and environmental factors. The TORCH infections (toxoplasma, rubella, cytomegalovirus, herpes simplex virus) and multiple syndromes (Mackel‐Gruber syndrome, Walker‐Warburg syndrome, Amniotic band syndrome, etc.) have been implicated in the causation of NTDs.^1^

Embryologically, the rostral neuropore closes on the 25th day after conception, and the caudal neuropore closes 2 days later. Periconceptional folic acid supplementation is recommended to prevent these closures and is commonly practiced in antenatal care. However, the relationship between maternal folic acid supplementation and the development of encephalocele is still not clear. Women who take 4 mg of folic acid per day before pregnancy and through the 12th week of gestation will only experience a 72% protective effect.^2^

## AUTHOR CONTRIBUTIONS

**Binod Paudel:** Conceptualization; data curation; formal analysis; funding acquisition; investigation; methodology; project administration; resources; software; supervision; validation; visualization; writing – review and editing. **Gopal K. Yadav:** Conceptualization; data curation; formal analysis; funding acquisition; investigation; methodology; resources; software; supervision; validation; visualization; writing – original draft; writing – review and editing.

## FUNDING INFORMATION

None.

## CONFLICT OF INTEREST STATEMENT

The authors declare no conflict of interest.

## CONSENT

Written informed consent was obtained from the patient's mother to use the data and pictures, and publish the report in accordance with journal's patient consent policy.

## Data Availability

The data that support the findings of this study are available on request from the corresponding author.
